# Household transmission of *Neisseria meningitidis* in the meningitis belt

**DOI:** 10.1016/S2214-109X(16)30292-3

**Published:** 2016-12

**Authors:** Sarah A Meyer, Paul A Kristiansen

**Affiliations:** Meningitis and Vaccine Preventable Diseases Branch, US Centers for Disease Control and Prevention, Atlanta, GA 30329, USA (SAM); and Norwegian Institute of Public Health, Oslo, Norway (PAK)

The epidemiology of *Neisseria meningitidis* is dynamic, with risk of meningococcal disease varying widely by region and depending on a confluence of host, organism, and environmental factors. Because transmission of *N meningitidis* results mainly in asymptomatic carriage, evaluation of oropharyngeal carriage can be helpful to understand the epidemiology and transmission of *N meningitidis* and, in turn, develop strategies for the prevention and control of meningococcal disease. The bacterium is transmitted through respiratory droplets and close contact, with transmission increasing in crowded settings such as military camps, universities, and schools.^1^ Household contacts of patients with meningococcal disease have been shown to be at increased risk of meningococcal carriage and disease in developed countries, where incidence of meningococcal disease is low and outbreaks infrequent. However, less is known about household transmission dynamics of *N meningitidis* in the unique epidemiological context of the meningitis belt of sub-Saharan Africa, which is characterised by high rates of endemic disease, annual outbreaks, and periodic large-scale epidemics, historically due to serogroup A meningococci.^2^

In *The Lancet Global Health*, Caroline Trotter and colleagues^3^ describe the importance of household transmission of *N meningitidis* in the meningitis belt using data from a series of cross-sectional meningococcal carriage surveys held across seven countries to describe meningococcal carriage and impact of a novel meningococcal serogroup A conjugate vaccine (MenAfriVac; Serum Institute of India PVT, Pune, India). Within the study the investigators recruited a subset of 184 households containing putative *N meningitidis* carriers due to any serogroup for longitudinal household carriage surveys carried out over 6 months. 133 households with confirmed index carriers were compared with 51 control households in which *N meningitidis* in the putative index carrier was ruled out by reference testing. 21% (152 of 739) of individuals within index carrier households subsequently acquired *N meninigitidis* compared with 9% (35 of 371) of individuals in control households. Due to a paucity of serogroup A carriers, the impact of MenAfriVac vaccination on carriage acquisition or loss within households could not be determined. Although the overall carriage acquisition rate was 2·4% per month (95% CI 1·6–4·0), rates among all age groups were four-to-five-times higher in households with an index carrier. Overall, the mean duration of carriage was 3·4 months (2·7–4·4). Index carriers were most likely to be adolescents, with a median age of 12 years, and children younger than 5 years were most likely to acquire carriage. In index carrier households, most individuals that subsequently developed carriage acquired the same or a similar strain as the index carrier, providing evidence for within-household transmission, although external acquisition was also noted. Further analysis of the strains with next-generation sequencing will be useful to further differentiate transmission within households versus external acquisition.

Since the progressive introduction of the meningococcal serogroup A conjugate vaccine in meningitis belt countries via mass vaccination campaigns of 1–29 year olds starting in 2010, a remarkable effect of the vaccine has been observed.^4^ Similar to other conjugate vaccines, MenAfriVac has demonstrated the ability to markedly reduce serogroup A *N meningitidis* carriage prevalence and generate herd immunity, likely contributing to the near-elimination of serogroup A disease in vaccinated areas.^5,6^ However, epidemics due to other serogroups, such as the 2015 serogroup C epidemics in Niger and Nigeria,^7,8^ continue to occur. Thus, additional strategies for the control of meningococcal disease are needed.

The findings of Trotter and colleagues^3^ provide further insight into transmission dynamics of *N meningitidis* within households in the meningitis belt. However, the low sensitivity rate of oropharyngeal swabbing (estimated as 57·8% [95% CI 53·5–62·0] in this study) is a limitation. Nevertheless, results of this evaluation along with surveillance data suggest that targeting school-age children and adolescents for vaccination with conjugate vaccines could provide maximum benefit in terms of direct protection and generation of herd immunity. Further household carriage evaluations specifically carried out during epidemics are needed to assess the relative importance of household transmission in the setting of widespread community transmission. Antibiotic chemoprophylaxis of household members of meningococcal disease cases is recommended in the meningitis belt outside of outbreaks,^9^ although is rarely practiced due to resource and logistical constraints. Even though no known cases of meningococcal disease were reported in households participating in the study from Trotter and colleagues, the increased rate of subsequent carriage in index households supports this recommendation and efforts to improve its uptake. Additional evaluation of carriage among household contacts of a meningococcal case in both outbreak and non-outbreak settings would provide additional data to inform antibiotic chemoprophylaxis recommendations in the meningitis belt.

Despite the early successes of the MenAfriVac vaccine, endemic disease and epidemics due to serogroups C, W, and X continue to occur. Additional carriage evaluations will be helpful to continue to monitor the impact of MenAfriVac on serogroup A carriage as well as to support the development and evaluation of additional strategies for the control of meningococcal disease in this region.
